# Supplementary material for: PolyCL: contrastive learning for polymer representation learning via explicit and implicit augmentations
Source: Digit Discov. 2024 Nov 28;4(1):149–60. doi: 10.1039/d4dd00236a (PMC11616009; doi:10.1039/d4dd00236a)
Supplement: DD-004-D4DD00236A-s001 [file DD-004-D4DD00236A-s001.pdf]

Supplementary Information (SI) for Digital Discovery.
This journal is © The Royal Society of Chemistry 2024

Supporting Information: PolyCL: Contrastive Learning for Polymer Representation Learning via Explicit and Implicit Augmentations

Jiajun Zhou,[†] Yijie Yang,[†] Austin M. Mroz,[†,‡] and Kim E. Jelfs[*,†]

†*Department of Chemistry, Molecular Sciences Research Hub, Imperial College London, White City Campus, Wood Lane, London, W12 0BZ, U.K.*

‡*I-X Centre for AI in Science, Imperial College London, White City Campus, Wood Lane, London, W12 0BZ, U.K.*

E-mail: k.jelfs@imperial.ac.uk

Contents

S1 Downstream Datasets **S-3**

S2 Detailed Neural Network Modules **S-4**

S3 Transfer Learning Performance **S-5**

S4 Fine-Tune Performance of Self-Supervised Learning Models **S-6**

S5 Pre-trained Model Comparison **S-7**

S6 Results of TSNE on Downstream Datasets **S-8**

S7 Training Details of Supervised Models **S-9**

S7.1 Random Forest with ECFP S-9

S7.2 XGBoost with ECFP S-9

S7.3 Neural Network with ECFP S-9

S7.4 Gaussian Process and Neural Network with Polymer Genome S-10

S7.5 Graph Convolutional Network S-10

References **S-11**

S1 Downstream Datasets

Table S1: Downstream datasets sourced from Xu *et al.*[S1]

Dataset	Property	Size	Unit
Egc	bandgap(chain)	3380	eV
Egb	bandgap(bulk)	561	eV
Eea	electron affinity	368	eV
Ei	ionisation energy	370	eV
Xc	crystallisation tendency	432	%
EPS	dielectric constant	382	-
Nc	refractive index	382	-

S2 Detailed Neural Network Modules

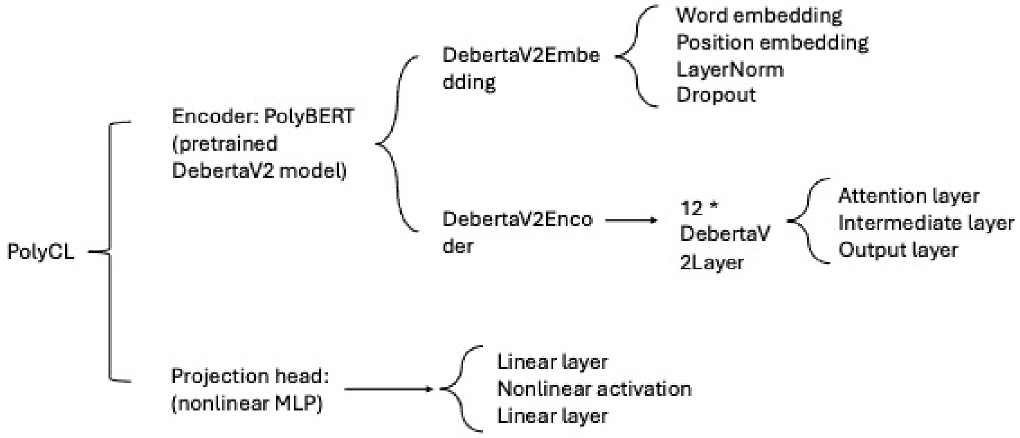

Figure S1: Detailed neural network modules of PolyCL

S3 Transfer Learning Performance

Here, we report the average root mean square error of the transfer learning task, where only the prediction head was fine-tuned and the pre-training model was frozen.

Table S2: The average root mean square error on the unseen validation datasets with Five-Fold Cross-Validation. #Params is the parameter count in the model.

Model information		Datasets						
Model	#Params	Eea	Egb	Egc	Ei	EPS	Nc	Xc
RF_{ECFP}	-	0.4295	0.7163	0.5623	0.4978	0.6220	0.1181	17.7347
XGB_{ECFP}	-	0.4342	0.7184	0.5674	0.5151	0.6336	0.1173	18.5263
NN_{ECFP}	264K	0.4095	0.6989	0.5324	0.4835	0.5543	0.1046	18.2696
GP_{PG} [S2]	-	0.32	0.55	0.48	0.42	0.53	0.10	24.42
NN_{PG} [S2]	-	0.32	0.57	0.49	0.45	0.54	0.10	20.74
GCN	70K	0.4146	0.8605	0.7009	0.5040	0.6472	0.1655	19.3743
GIN	218K	0.3717	0.7854	0.6663	0.4590	0.6197	0.1456	18.5009
TransPolymer[S1]	82.1M	0.3446	0.6266	0.5498	0.4450	0.5475	0.1033	17.4086
PolyBERT[S3]	25.2M	0.3272	0.6636	0.5448	0.4672	0.5326	0.1063	17.6989
PolyCL	25.2M	0.3265	0.6458	0.5329	0.4180	0.5111	0.0933	18.1747

S4 Fine-Tune Performance of Self-Supervised Learning Models

Here, the average R^2 values and root mean square errors of the fine-tuning task are shown, where both the pre-trained model and prediction head were fine-tuned.

Table S3: The average of R^2 on the unseen validation datasets with Five-Fold Cross-Validation on self-supervised models. #Params is the parameter count in the model. The boldings indicate the best results.

Model information		Datasets							
Model	#Params	Eea	Egb	Egc	Ei	EPS	Nc	Xc	Avg.R^2
TransPolymer[S1]	82.1M	0.9179	**0.9365**	0.9177	0.8278	0.7966	0.8674	0.4500	0.8163
PolyBERT[S3]	25.2M	**0.9365**	0.9243	0.9163	0.8359	**0.8081**	0.8653	0.4428	**0.8185**
PolyCL	25.2M	0.9317	0.9273	**0.9186**	**0.8491**	0.8038	**0.8699**	0.4250	0.8179

Table S4: The average root mean square error on the unseen validation datasets with Five-Fold Cross-Validation. #Params is the parameter count in the model.

Model information		Datasets						
Model	#Params	Eea	Egb	Egc	Ei	EPS	Nc	Xc
TransPolymer[S1]	82.1M	0.3031	0.4901	0.4480	0.4052	0.5014	0.0859	17.3788
PolyBERT[S3]	25.2M	0.2659	0.5304	0.4517	0.3972	0.4850	0.0875	17.6082
PolyCL	25.2M	0.2757	0.5243	0.4457	0.3799	0.4923	0.0858	17.8229

S5 Pre-trained Model Comparison

Table S5: Comparison of pre-trained Polymer Foundation Models

Model	Training Set Size	Training Strategy	#Params
TransPolymer	5M	MLM	82.1M
PolyBERT	80M	MLM	25.2M
PolyCL	80M + 1M[1]	Contrastive Learning	25.2M

[1] PolyCL has PolyBERT as its pre-trained prior. PolyCL is trained with 1M datapoints. MLM indicates masked language modelling. #Params is the parameter count for each model.

S6 Results of TSNE on Downstream Datasets

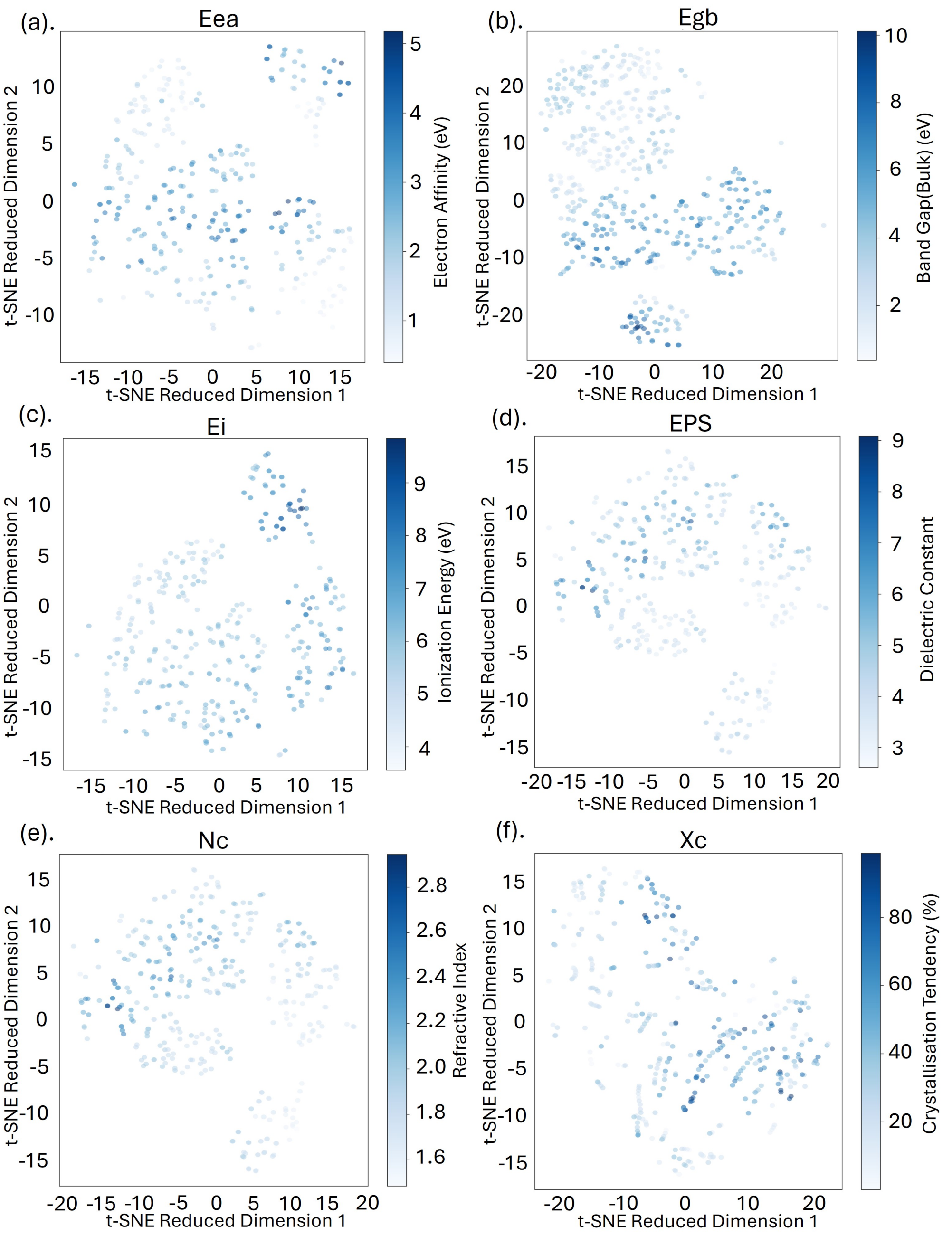

Figure S2: t-SNE dimensional reduction analysis of the polymer representation space learnt by PolyCL. Visualisation of the continuous representation of polymer repeating units. Each figure on the downstream dataset is coloured by the corresponding value of the property in the downstream dataset. (a) Eea dataset coloured by electron affinity. (b) Egb dataset coloured by bandgap (bulk). (c) Ei dataset coloured by ionisation energy (d) EPS dataset coloured by dielectric constant (e) Nc dataset coloured by refractive index (f) Xc dataset coloured by crystallisation tendency

S7 Training Details of Supervised Models

A five-fold cross-validation process was applied to each algorithm. The fold average of all R^2 and $RMSE$ values on each unseen validation set were taken to evaluate the model performance.

S7.1 Random Forest with ECFP

We used RDKit[S4] to transfer the polymer-SMILES to 512-bit ECFP fingerprints. The scikit-learn package[S5] was used to construct the random forest regressor and hyperparameters were tuned manually. The final random forest model we used is:

```
sklearn.ensemble.RandomForestRegressor
(bootstrap=False,max_features='sqrt',random_state=72)
```

where all other parameters in the above implementation remain the default parameters.

S7.2 XGBoost with ECFP

We constructed 512-bit ECFP fingerprints as shown in Section S7.1. The XGBoost[S6] package was used to construct our xgboost model. After tuning, the final model is:

```
xgboost.XGBRegressor
(max_depth=6,min_child_weight=3,colsample_bytree=0.8,random_state=72)
```

where all other parameters in the above implementation remain the default parameters.

S7.3 Neural Network with ECFP

We obtained the same ECFP fingerprints as shown in Section S7.1. We employed a simple two-layered MLP with a dropout ratio of 0.1. As an early stopping mechanism, a patience value of 100 was used with the epoch size set to a large value. During training, the batch size was set to 16 and the learning rate was 0.001.

S7.4 Gaussian Process and Neural Network with Polymer Genome

The performances are directly taken from the originally reported results by Kuenneth *et al.*[S2]

S7.5 Graph Convolutional Network

We abstracted polymers to two-dimensional undirected molecular graphs. We follow the featurisation method used by Hu *et al.*[S7] to encode nodes and edges to the graph representation using feature sets. Features are extracted using RDKit.[S4] The connecting point in polymer data is marked as a special node indexed by 0 in the node set.

Table S6: Feature sets for nodes and edges in molecular graphs.

**Component**	**Feature**	**Feature Set Details**
Node	Atomic number	[0, 119]
Node	Chirality	unspecified, tetrahedral CW, tetrahedral CCW, other
Edge	Bond type	single, double, triple, aromatic
Edge	Bond direction	none, end-upright, end-downright

The nodes and edges were integrated by using PyTorch Geometric[S8] to construct molecular graphs.

A molecular graph $G = (V, E)$ is constructed by collections of nodes V and edges V, where the node features are denoted by X_v for $v \in V$. GNN layers learn to create embeddings h_v at the node level, by aggregating the features X_v from neighbouring nodes. Consequently, the node embedding after k-th layers encompasses the information within its k-hop neighbourhood. The k-th layer of a GNN can be described by:

$$\begin{aligned} a_v^{(k)} &= \text{AGGREGATE}^{(k)}\left(\left\{h_u^{(k-1)} : u \in \mathcal{N}(v)\right\}\right) \\ h_v^{(k)} &= \text{COMBINE}^{(k)}\left(h_v^{(k-1)}, a_v^{(k)}\right) \end{aligned} \tag{1}$$

In practice here, we used two different architectures of $\text{AGGREGATE}^{(k)}$ to construct GNNs to process polymer graphs. In graph convolutional networks (GCN), element-wise

mean pooling is proposed.[S9] Therefore, the update rule can be summarised as:

$$h_v^{(k)} = \mathrm{ReLU}\left(W^{(k)} \cdot \mathrm{MEAN}\left(\{h_u^{(k-1)} : u \in \mathcal{N}(v) \cup \{v\}\}\right)\right) \tag{2}$$

Alternatively, graph isomorphism networks (GIN)[S10] use a sum aggregation function and non-linear transformation by an MLP:

$$h_v^{(k)} = \mathrm{MLP}^{(k)}\left(\left(1 + \epsilon^{(k)}\right) \cdot h_v^{(k-1)} + \sum_{u \in \mathcal{N}(v)} h_u^{(k-1)}\right) \tag{3}$$

For the final layer node representation $h_v^{(k)}$, the READOUT function is used to aggregate node features to obtain the graph representation:

$$h_G = READOUT(h_v^{(k)} | v \in G) \tag{4}$$

For both types of graph neural networks, GCN and GIN, we used the same set of hyperparameters except for the aggregation process inherently defined differently as shown in Equation 2 and 3. We used 3 layered GNN with a dropout rate of 0.1. The node and edge attributes were embedded in 128-dimensional space. The supervised training process was set to last for 100 epochs. An early stopping mechanism was implemented with a patience value of 50 epochs.

References

(S1) Xu, C.; Wang, Y.; Barati Farimani, A. TransPolymer: a Transformer-based language model for polymer property predictions. *npj Comput. Mater.* **2023**, *9*, 64.

(S2) Kuenneth, C.; Rajan, A. C.; Tran, H.; Chen, L.; Kim, C.; Ramprasad, R. Polymer informatics with multi-task learning. *Patterns* **2021**, *2*, 100238.

(S3) Kuenneth, C.; Ramprasad, R. polyBERT: a chemical language model to enable fully machine-driven ultrafast polymer informatics. *Nat. Commun.* **2023**, *14*, 4099.

(S4) Landrum, G. Rdkit documentation. *Release* **2013**, *1*, 4.

(S5) Pedregosa, F.; Varoquaux, G.; Gramfort, A.; Michel, V.; Thirion, B.; Grisel, O.; Blondel, M.; Prettenhofer, P.; Weiss, R.; Dubourg, V.; Vanderplas, J.; Passos, A.; Cournapeau, D.; Brucher, M.; Perrot, M.; Duchesnay, E. Scikit-learn: Machine Learning in Python. *J. Mach. Learn. Res.* **2011**, *12*, 2825–2830.

(S6) Chen, T.; Guestrin, C. XGBoost: A Scalable Tree Boosting System. Proceedings of the 22nd ACM SIGKDD International Conference on Knowledge Discovery and Data Mining. New York, NY, USA, 2016; pp 785–794.

(S7) Hu, W.; Liu, B.; Gomes, J.; Zitnik, M.; Liang, P.; Pande, V.; Leskovec, J. Strategies for pre-training graph neural networks. *arXiv* **2019**, preprint, arXiv:1905.12265.

(S8) Fey, M.; Lenssen, J. E. Fast graph representation learning with PyTorch Geometric. *arXiv* **2019**, preprint arXiv:1903.02428.

(S9) Kipf, T. N.; Welling, M. Semi-supervised classification with graph convolutional networks. *arXiv* **2016**, preprint, arXiv:1609.02907.

(S10) Xu, K.; Hu, W.; Leskovec, J.; Jegelka, S. How powerful are graph neural networks? *arXiv* **2018**, preprint, arXiv:1810.00826.
